# SG-APSIC1123: Diagnostic and infection prevention and control (IPC) performance of rapid polymerase chain reaction (PCR) compared to conventional culture PCR methods for detecting carbapenemase-producing organisms (CPOs)

**DOI:** 10.1017/ash.2023.78

**Published:** 2023-03-16

**Authors:** Xiaowei Huan, Kyaw Zaw Linn, Sharifah Farhanah, Clara Chong Hui Ong, Pei Yun Hon, Yan Sun, Weixiang Lian, Janis Wanning Loh, Angela Chow, Benda Sze Peng Ang, Janice Wai Yeng Leong, Partha P De, Oon Tek Ng, Marimuthu Kalisvar, Shawn Vasoo

**Affiliations:** National Centre for Infectious Diseases, Singapore; National Public Health & Epidemiology Unit, National Centre for Infectious Diseases, Singapore; Infectious Disease Research and Training Office, National Centre for Infectious Diseases, Singapore; Infectious Disease Research and Training Office, National Centre for Infectious Diseases, Singapore; Infectious Disease Research Laboratory, National Centre for Infectious Diseases, Singapore; Health Service and Outcomes Research, National Healthcare Group Pte Ltd, Singapore; Clinical Epidemiology, National Centre for Infectious Diseases, Singapore; Clinical Epidemiology, National Centre for Infectious Diseases, Singapore; Clinical Epidemiology, National Centre for Infectious Diseases, Singapore; Infectious Diseases, Tan Tock Seng Hospital, Singapore; Department of Laboratory Medicine, Tan Tock Seng Hospital, Singapore; Department of Laboratory Medicine, Tan Tock Seng Hospital, Singapore; Infectious Diseases, Tan Tock Seng Hospital, Singapore; Infectious Diseases, Tan Tock Seng Hospital, Singapore; Infectious Diseases, Tan Tock Seng Hospital, Singapore

## Abstract

**Objectives:** In this study, we compared the performance of a rapid polymerase chain reaction (PCR) method in detecting carbapenemase-producing organisms (CPOs) and its impact on infection prevention and control (IPC) measures compared with a culture PCR method. **Methods:** All patients requiring CPO screening were included. Rectal swabs were collected with double rayon swabs (Copan 139C). They were simultaneously analyzed for the presence of CPOs using rapid PCR assay (Xpert Carba-R assay, Cepheid, Sunnyvale, CA) and a culture–PCR method (ChromID CARBA-SMART, bioMerieux, Marcy-l’Etoile, France). For CARBA-SMART, only colored colonies (ie, Enterobacterales) were evaluated for CPOs according to the prevailing institutional protocol. We tracked time to CPO detection. Using CPO positivity from either the rapid PCR or the culture PCR method as the gold standard, we calculated the sensitivity and specificity of both tests. We calculated the number of epidemiologically linked contacts generated when the first test results were known. We prospectively followed the ward census to identify the putative additional number of contacts generated by the later known result. Contacts were patients who shared the same ward (with overlapping time) as the CPO patients. **Results:** Between April 2019 and June 2020, culture PCR method detected CPOs in 316 (1.3%) of 24,514 samples (*bla*OXA48, N = 211; *bla*NDM, N = 51; *bla*IMI, N = 21; *bla*IMP, N = 10; *bla*KPC, N = 9; mixed genotypes, N = 14). The rapid PCR test detected CPOs in 605(2.5%) of 24,514 samples (*bla*OXA48, N = 266; *bla*NDM, N = 161; *bla*IMP, N = 99; *bla*VIM, N = 29; *bla*KPC, N = 15; mixed genotypes, N = 35). The sensitivity of direct PCR and culture PCR methods were 94.2% (95% CI, 92.1%–95.8%) and 43.5% (95% CI, 39.6%–47.4%), respectively. Both tests had 100% specificity. The median times to detection for the rapid PCR and culture PCR methods were 3–4 hours and 4 days, respectively. Compared with rapid PCR, the culture PCR method generated additional 7,415 contacts when it also tested positive for CPOs and an additional 23,135 contacts when it tested negative for CPOs. **Conclusions:** In our study, the rapid PCR test was more sensitive, identified CPO faster, and generated fewer epidemiologically linked contacts than the culture PCR method.

